# Probabilistic Downscaling of Remote Sensing Data with Applications for Multi-Scale Biogeochemical Flux Modeling

**DOI:** 10.1371/journal.pone.0128935

**Published:** 2015-06-12

**Authors:** Paul C. Stoy, Tristan Quaife

**Affiliations:** 1 Department of Land Resources and Environmental Science, Montana State University, Bozeman, Montana, United States of America; 2 Department of Meteorology, University of Reading, Reading, United Kingdom; University of Calgary, CANADA

## Abstract

Upscaling ecological information to larger scales in space and downscaling remote sensing observations or model simulations to finer scales remain grand challenges in Earth system science. Downscaling often involves inferring subgrid information from coarse-scale data, and such ill-posed problems are classically addressed using regularization. Here, we apply two-dimensional Tikhonov Regularization (2DTR) to simulate subgrid surface patterns for ecological applications. Specifically, we test the ability of 2DTR to simulate the spatial statistics of high-resolution (4 m) remote sensing observations of the normalized difference vegetation index (NDVI) in a tundra landscape. We find that the 2DTR approach as applied here can capture the major mode of spatial variability of the high-resolution information, but not multiple modes of spatial variability, and that the Lagrange multiplier (*γ*) used to impose the condition of smoothness across space is related to the range of the experimental semivariogram. We used observed and 2DTR-simulated maps of NDVI to estimate landscape-level leaf area index (LAI) and gross primary productivity (GPP). NDVI maps simulated using a *γ* value that approximates the range of observed NDVI result in a landscape-level GPP estimate that differs by *ca* 2% from those created using observed NDVI. Following findings that GPP per unit LAI is lower near vegetation patch edges, we simulated vegetation patch edges using multiple approaches and found that simulated GPP declined by up to 12% as a result. 2DTR can generate random landscapes rapidly and can be applied to disaggregate ecological information and compare of spatial observations against simulated landscapes.

## Introduction

Upscaling estimates of ecosystem function from leaf to region to globe and downscaling remote sensing observations and general circulation model predictions to smaller scales remain basic research challenges across a wide range of Earth science disciplines. Scaling is a procedure that takes information at one scale in time and/or space and uses it to derive processes at another [1]. Following this definition, scaling inherently involves a transfer of information. Scaling in the Earth sciences is therefore related to Information Theory, the study of the quantification and transfer of information [2–5].

Information is quantified by its entropy [2], defined by its probability distribution (or density) function (pdf). Taking the Information Theory-based definition of scaling, downscaling involves finding some ‘hidden’ pdf at a higher sensor resolution, a finer model grain size, or some other smaller spatial scale depending on the topic of interest. Inferring an unknown pdf is an ill-posed problem in mathematics, and a common solution to this problem is to incorporate additional information using Tikhonov Regularization (TR, [6]). Applications of TR are scarce in ecological science, but are more common in the Earth sciences. A typical usage is to constrain retrievals of information from satellite data. Quaife and Lewis [7], for example, used TR to stabilize model parameter retrievals from satellite observations by applying the *a priori* assumption that the parameters should be smooth in time. Here, we use two-dimensional Tikhonov Regularization (2DTR) to demonstrate that applying the constraint that first differences should be small to a random field imposes spatial structure on downscaled information.

The generation of random fields has long been of interest across academic disciplines. Ecologists and hydrologists frequently use random (often called ‘neutral’) landscapes as a basis for comparison against observations [8–12]. Earth scientists generate random features to simulate partially observable entities such as clouds [13] or simulate subgrid land surface characteristics based on physical attributes like lateral heat transport [14]. Computer scientists use simulated surfaces to challenge optimization routines [15].

Most random field generation methods make certain assumptions regarding spectral [16], fractal [10], or hierarchical [9] structure of the field to be simulated. Such statistical attributes are valuable to include in a random landscape generator if known, but may be unknown. Random landscape generation in Ecology has tended to focus on simulating discrete classes [8] such as suitable or unsuitable habitat [11], rather than continuous variables that may be of interest for quantifying ecosystem functioning. Here, we use 2DTR to simulate continuous surfaces for the purpose of developing multi-scale estimates of ecosystem function assuming minimal prior information.

We explore the ability of 2DTR to downscale coarse-scale remote sensing data for the purpose of simulating fine-scale surface patterning, and provide an example by simulating landscape-level patterns of the normalized difference vegetation index (NDVI), leaf area index (LAI), and gross primary productivity (GPP) in tundra. We choose this example for a number of reasons. Arctic terrestrial ecosystems are an important component of the global C cycle [17] and control heat, water, and biogeochemical exchanges between biosphere, cryosphere and atmosphere in a rapidly changing climate [18]. LAI is nonlinearly related to observables like NDVI, and to important carbon cycling processes including the GPP [19], and bias due to Jensen’s Inequality results if average LAI is used in GPP estimation [20,21]. Tundra has the greatest spatial variability of LAI of any global ecosystem [22], and quantifying the statistics of LAI, not merely its magnitude, is important for estimating tundra CO_2_ flux [21,23]. The spatial statistics of LAI may also be important for unbiased estimates of GPP in tundra; for example, Fletcher et al. [24] found that GPP per unit LAI in the transition zones (henceforth called ‘edges’, although often called ‘ecotones’) between main vegetation patches is 20% to 40% lower than GPP per unit LAI at patch centers [25]. These results suggest that scaling GPP without incorporating vegetation edge effects may lead to bias in landscape level GPP estimates. We use the nonlinear relationships between NDVI, LAI, and GPP in tundra and the goal of reducing bias in GPP estimates as a motivation for simulating subgrid NDVI patterns using 2DTR.

We first describe 2DTR, then use 2DTR to simulate landscapes with the statistics and spatial patterning of fine scale (4 m) NDVI from a tundra ecosystem near Abisko, Sweden, beginning with aggregated NDVI on the coarse (250 m) spatial resolution of the moderate resolution imaging spectroradiometer (MODIS). We estimate subgrid LAI patterns from the simulated NDVI landscapes using nonlinear NDVI-LAI transfer functions [26,27]. GPP is then estimated by combining a validated tundra ecosystem model (PLIRTLE, Shaver et al. [19]) with micrometeorological observations to estimate landscape-level photosynthetic C uptake. Finally, we explore approaches for incorporating vegetation edge effects into the landscape-level GPP estimate [28], and discuss the limitations and opportunities of 2DTR in the context of other random field generators.

## Materials and Methods

### 2D Tikhonov Regularization

Given only a single measured value representing the mean of some distributed attribute of an extended point in space, i.e. the value of a remotely sensed pixel, the best estimate of its disaggregated form without additional information is that it is uniformly equal to the recorded value, a Dirac delta function. With knowledge of the minimum and maximum of its likely state and ignoring the fact that we know the mean, the best estimate of the underlying distribution is drawn from the uniform distribution bounded by logical extrema; i.e. an uninformed prior, often referred to as the default model, which is the distribution that maximizes information entropy in this case. For some applications this level of disaggregation may be sufficient. However, the true hidden probability distribution is likely between the Dirac delta and the uniform distribution. Inferring this probability distribution and corresponding spatial statistics of subgrid elements is a more complex problem and for this we adopt 2DTR [6].

We make the *a priori* assumption that subgrid elements are likely to be similar to those at adjacent subgrid pixels by specifying that first differences should tend toward zero. The remaining problem is to define the strength of this assumption, for which additional information may be available. In the case of spatial ecology and remote sensing, descriptive statistics such as the total variance, semivariogram range, or characteristic patch size may be known or can be approximated [29]; these represent additional information constraints on the unknown pdf to be estimated. In other words, we may have some summary and/or spatial statistics to add to the null assumption that adjacent pixels are similar and to the logical assumption that values are bounded.

The form of the regularization we propose to provide spatial disaggregation is given by

$$\alpha \prime ={\left(I+\gamma^{2}B^{T}B\right)}^{-1}\alpha \frac{\sigma^{2}}{\psi \left(\gamma^{2}\right)}-\mu_{\alpha }+\mu_{\alpha \prime },$$ (1)

where *α* is a matrix of draws from a probability distribution, *μ* is its aggregate mean, *B* is an expression of the required constraint that neighboring elements of the subgrid are similar, *I* is the identity matrix, *σ* ^2^ is the total variance of *α*, and *γ* is a Lagrange multiplier. *α* can take any distribution, but here we choose the uniform distribution *U*(min,max) to represent an uninformed case with no prior knowledge of the underlying distribution. The choice of min = 0 and max = 1 here corresponds to reasonable bounds for the NDVI of a terrestrial surface, but is otherwise arbitrary as the data is scaled according to the normalizing term *Ψ*(*γ* ^2^), which is equal to the variance of (*I*+*γ* ^2^ *B* ^T^ *B*)^−1^ *α*.

The system in (1) sets *α = α*' subject to the constraint *Bα*' = *z* where z is a vector of zeros of the same length as *α*. The matrix *B* is formulated to provide the specified constraint. In the case discussed here it constrains the result to have a first difference of zero in the cardinal directions of the image space, i.e. it assumes that adjacent pixels are likely similar to the value of a given pixel. *γ* imposes the strength of this assumption. If the value of *γ* is very large, then *Bα*'→0 and all the elements of *α*' will be constrained to be close to *μ*. As *γ*→0, then *α*'→*α*, the random draw. *γ* is, in effect, a balancing term between these two possible solutions: a wholly random draw or a uniform surface whose elements take the value of *μ*.

NDVI observations from the high resolution 4 m data set from Abisko, Sweden chosen for this analysis have a mean (*μ*) of 0.54 and a variance (*σ* ^2^) of 0.009 [21]. Given this knowledge, a synthetic landscape representing and NDVI map can be created using 2DTR following these steps:

1. Consider a spatial domain and give it the dimensions of a MODIS NDVI pixel, 250 × 250 m in this example although the size of the domain strictly speaking does not matter. Say that this pixel has a NDVI of 0.54 and represents Arctic tundra.
2. Fill this pixel with a regular grid of subpixels with a length scale of 4 m, approximately the size of a characteristic vegetation patch in the Abisko tundra ecosystem [30,31], although any size smaller than the pixel suffices.
3. Let these subpixels take uniform random values drawn from *α* = *U*(0,1) to represent the range that NDVI values of the water-free terrestrial surface are likely to take.
4. Add information about the mean (*μ* = 0.54) and variance (*σ* ^2^ = 0.009), if known, to eq (1).
5. Assume that neighboring subpixels have similar values expressed via the two-dimensional first difference matrix *B*.
6. Constrain the strength of this assumption via the value of the Lagrange multiplier (*γ*) asking, effectively, how smooth is the surface?

An advantage of the 2DTR technique over other similar scene generating methods is that the inverse term (*I*+*γ* ^2^ *B* ^T^ *B*)^−1^ can be stored once calculated and the large numbers of scenes can be simulated quickly using randomly generated values of *α*. MATLAB code in support of this example is provided in the Supporting Information available on Montana State University Scholarworks at doi.org/10.15788/M21598.

### Gross primary productivity modeling

PLIRTLE [19] is a simple model that consistently explains some 75% of the variability of the eddy covariance or chamber-measured net ecosystem exchange of CO_2_ (NEE) in pan-Arctic ecosystems during the growing season [23,32]. PLIRTLE models GPP as a function of photosynthetically active photon flux density (PPFD) and LAI following the aggregated canopy model of Rastetter et al. [33]:

$$\text{GPP}=-\frac{P_{\text{max}}}{k}\text{ln}\left(\frac{P_{\text{max}}+E_{o}PPFD}{P_{\text{max}}+E_{o}PPFDe^{-k\text{LAI}}}\right).$$ (2)

We use parameter values from a pan-arctic parameterization of PLIRTLE [19] where the light-saturated photosynthetic rate *P*_max_ is 15.831 *μ*mol m^-2^ leaf s^-1^, the initial slope of the light response curve *E*_*o*_ is 0.036 *μ*mol CO_2_ *μ*mol photons^-1^, and the Beer’s Law extinction coefficient *k* is set to 0.5. Meteorological input is the same as used in Stoy et al. [21,30] for the June-July 2007 period from the Abisko Scientific Research Station, and is meant to approximate the growing season at Abisko.

LAI for PLIRTLE was estimated using the relationship with NDVI described by van Wijk and Williams [26] noting the adjustment discussed in [30]:

$$\text{LAI}=0.00067e^{9.237\text{NDVI}}$$ (3)

NDVI was calculated from an Azimuth Systems AZ-16 Airborne Thematic Mapper (ATM) overflight on 17^th^ July, 2005 over a tundra-dominated landscape near Abisko, Sweden investigated in previous studies [21,27,30,31,34]. These 4 m grid cells represent the finest spatial grain observed in the remotely-sensed NDVI that, upon conversion to LAI (eq 3) and modeling using PLIRTLE (eq 2), we take to be GPP for the study domain for the purposes of comparison. The study domain is near an intensive research site of the ABACUS-IPY project, comprising an eddy covariance tower [35] and other experimental measurements [36] to link process-level ecological studies in arctic ecosystems using multi-scale observations. No specific permissions were required for conducting these remote sensing activities.

### Approaches for simulating vegetation patch edges

We test four different methods for simulating vegetation patch edges and discuss their implications for downscaling the spatial distribution of GPP in tundra. Simulated NDVI is taken to represent patches of different vegetation types, a reasonable assumption given the relationship between NDVI and LAI and the differences in LAI among tundra vegetation patches [31]. The first (Method 1) simply starts at the mean NDVI value and selects pixels with similar NDVI until 30% of all pixels are reached and are denoted ‘edge’. Method 2 calculates the rate of change of NDVI (the slope) and assigns edge to the 30% of the pixels that have the highest slope. Method 3 is similar but calculates the rate of change of slope and likewise assigns edge to the 30% of the pixels that have the highest rate of change. Method 4 rounds each pixel of the NDVI map to the nearest integer, and denotes pixels adjacent to the boundary between 0 and 1 to be edge; for simulated landscapes with *γ* = 10^0.85^ (see Results) this happens to comprise some 30% of all pixels. Pixels that include edge were then simply multiplied by 0.7 to represent the mean 30% reduction in GPP found by Fletcher et al. [24].

## Results

### Inferring surface patterns using Tikhonov Regularization

**Fig 1:**
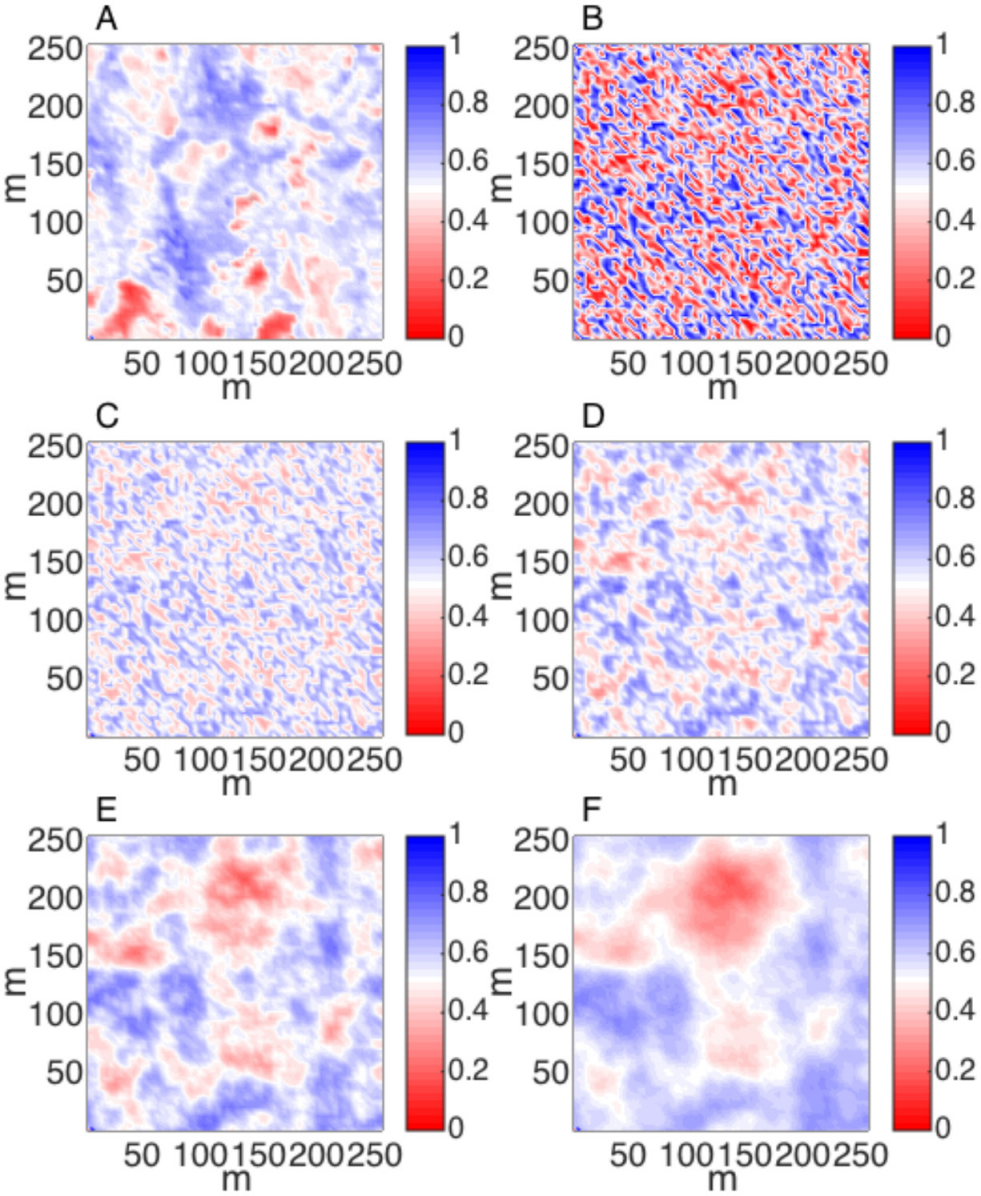
(A) Normalized difference vegetation index (NDVI) derived from advanced thematic mapper output in a tundra landscape near Abisko, Sweden after [21]. (B) A random uniform distribution of pixels, corresponding to *α* in eq (1), at the same spatial grain (4 m) as subplot (A). (C-F) Simulated distributions of NDVI by constraining (B) using 2D Tikhonov Regularization (eq 1) with the Lagrange multiplier (*γ*) equal to 0.1 (C), 1 (D), 10 (E) and 100 (F).

The observed NDVI map [21], a fully random uniform NDVI grid, and regularizations of that random grid using four different values of *γ* specifying the same mean (*μ* = 0.54) and variance (*σ* ^2^ = 0.009) of observed NDVI are shown in Fig 1. Note that the synthetic landscape with *γ* = 10 is visually most similar to the observed NDVI, the range of which is 47.7 m, or nearly twelve 4 m pixels.

**Fig 2:**
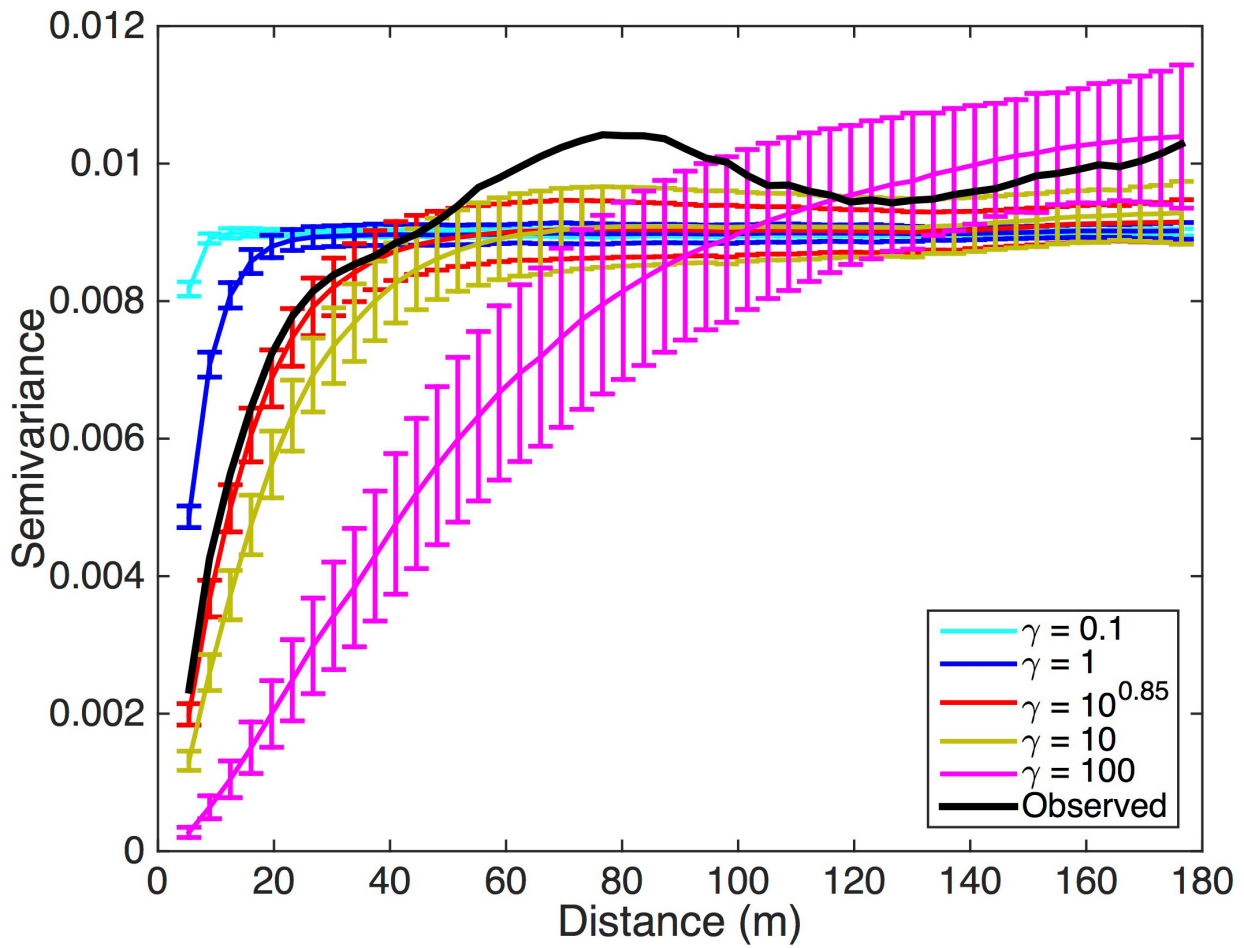
Experimental semivariograms fit to the observed NDVI map shown in Fig 1A, and the mean and variance of semivariograms from one hundred simulations of NDVI maps created using the two-dimensonal Tikhonov Regulariazation (2DTR) procedure for different values of the Lagrange multiplier *γ* (eq 1).

**Fig 3:**
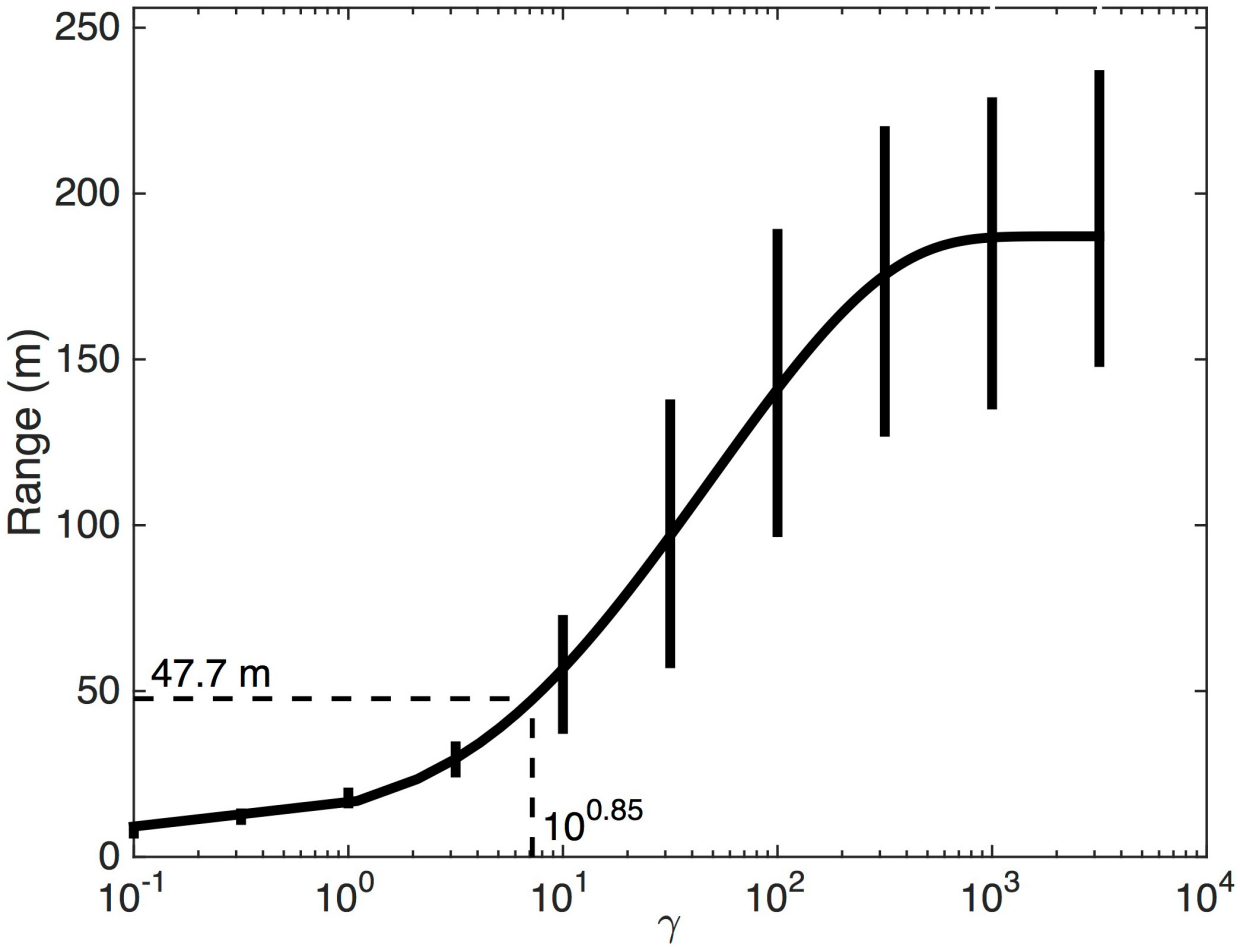
The mean and standard deviation of the semivariogram range for synthetic NDVI maps produced by applying 2DTR using different values of the Lagrange multiplier, *γ*. One hundred iterations for each value of *γ* were chosen to obtain representative statistics. The range of the observed NDVI image (47.7 m) and corresponding *γ* estimate (10^0.85^) are shown.

Semivariograms that correspond to the observed NDVI image and 100 iterations of the 2DTR-simulated NDVI maps are shown in Fig 2. The mean and standard deviation of parameters that result from fitting semivariograms to these 100 maps with a spherical model are displayed in Table 1. Fig 3 represents the relationship between different values of *γ* and the semivariogram range calculated using 100 iterations of simulated NDVI at multiple values of *γ*. The relationship approximates a generalized logistic function, and we fit such a function using nonlinear least squares to estimate the value of *γ*, approximately 10^0.85^, that corresponds to the range of observed NDVI, 47.7 m. We retain this value of *γ* for simulating landscape-level GPP in further examples.

**Table 1:** Statistics of experimental semivariograms fit to surfaces simulated using the 2DTR approach with different values of the Lagrange multiplier *γ*.

	Range (m)	Sill	Nugget (m)
*γ* = 0.1	10.4 ± 1.1	0.0027 ± 0.0017	0.0067 ± 0.0012
*γ* = 1	17.7 ± 1.4	0.0072 ± 0.0004	0.0018 ± 0.0004
*γ* = 10	52.5 ± 12.9	0.0086 ± 0.0006	0.0005 ± 0.0006
*γ* = 100	144.0 ± 46.6	0.010 ± 0.0009	0.0001 ± 0.0003
Observed	47.7	0.0077	0.0021

Values represent the mean ± the standard deviation of 100 iterations of the 2DTR procedure at each *γ*.

**Fig 4:**
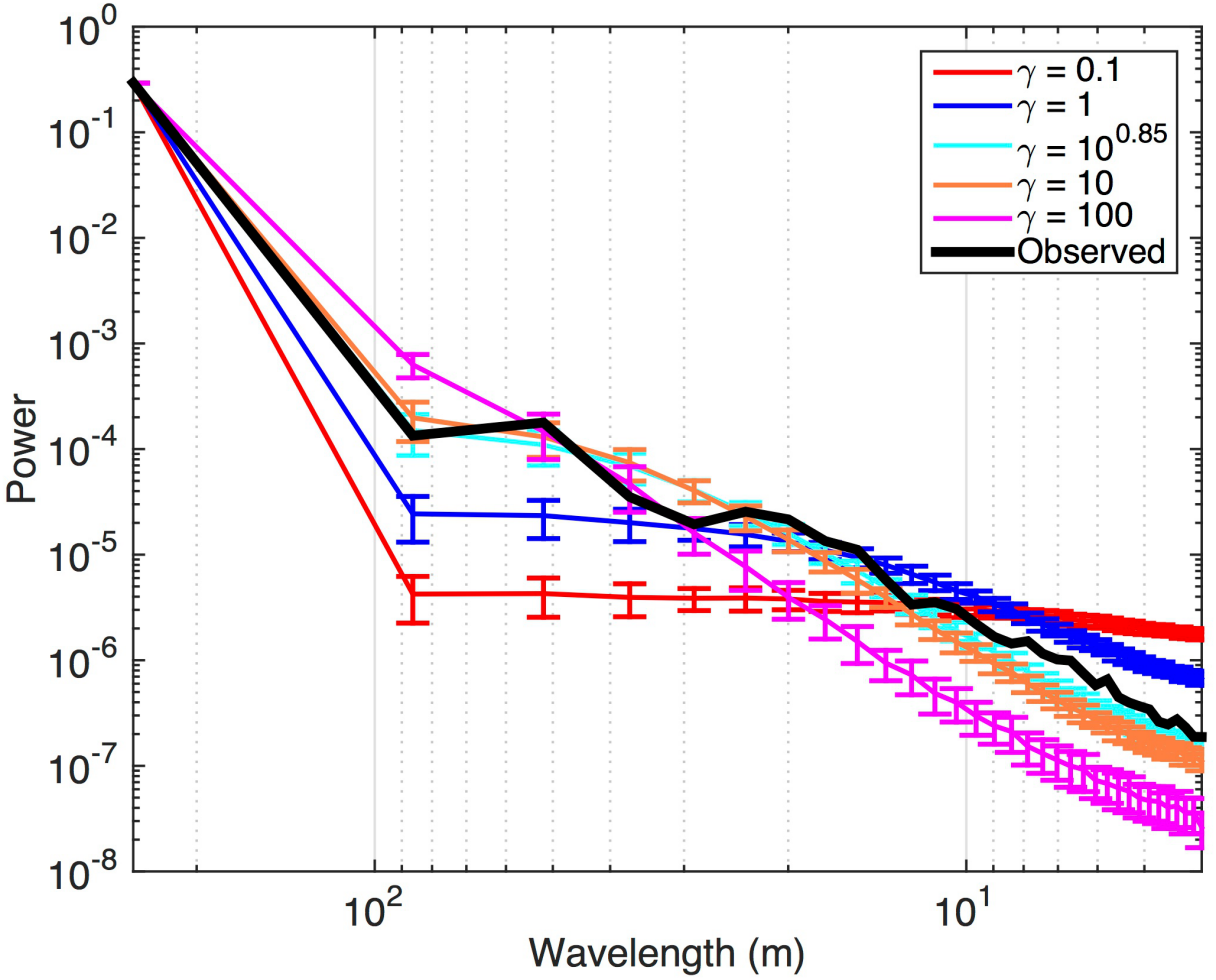
Radially-averaged power spectra for the observed NDVI image and 100 iterations of synthetic images generated by 2DTR for different values of the Lagrange multiplier *γ*.

From Fig 2 it is apparent that the semivariogram of the observed NDVI follows a more complicated spatial pattern than those of the simulated landscapes. The observed landscape contains more or less power at certain spatial frequencies than the 2DTR simulations with *γ* = 10^0.85^ is able to simulate, as also evidenced by the radially-averaged power spectra displayed in Fig 4. Specifically, the power spectrum of observations is more energetic than that of the 2DTR with *γ* = 10^0.85^ at frequencies between *ca*. 15 and 20 m, and is less energetic at frequencies between *ca*. 30 to 40 m. The 2DTR with *γ* = 10^0.85^ does however capture the dominant mode of spatial variability of the NDVI image between *ca*. 50 m–90 m. In other words, the random maps generated by 2DTR as applied here are able to encompass characteristic ranges (Figs 2 and 3) and frequencies (Fig 4), but not multiple modes of spatial variability. The question remains if the 2DTR maps that best simulate observed semivariogram range are likewise able to effectively simulate patterns in landscape-level GPP despite mismatches with observations at higher spatial frequencies.

### Simulating landscape-level gross primary productivity

A major motivation for simulating surface features is the finding that tundra vegetation patch edges have lower GPP than expected for a given value of LAI [24]. Shoot growth was found to be greater in the transition zone between vegetation patches [28], but GPP per unit LAI was some 20%-40% lower than the relationships at patch center [24] that was used for the parameterization of PLIRTLE [19,25]. Fletcher et al. [24] also found that patch edges encompass some 30% of the tundra landscape in Abisko and suggested that incorporating edge effects is critical for accurate upscaled estimates of GPP. We adopt this 30% edge criteria for the present study, and note the definition of edge will differ for different ecosystems and/or applications.

**Fig 5:**
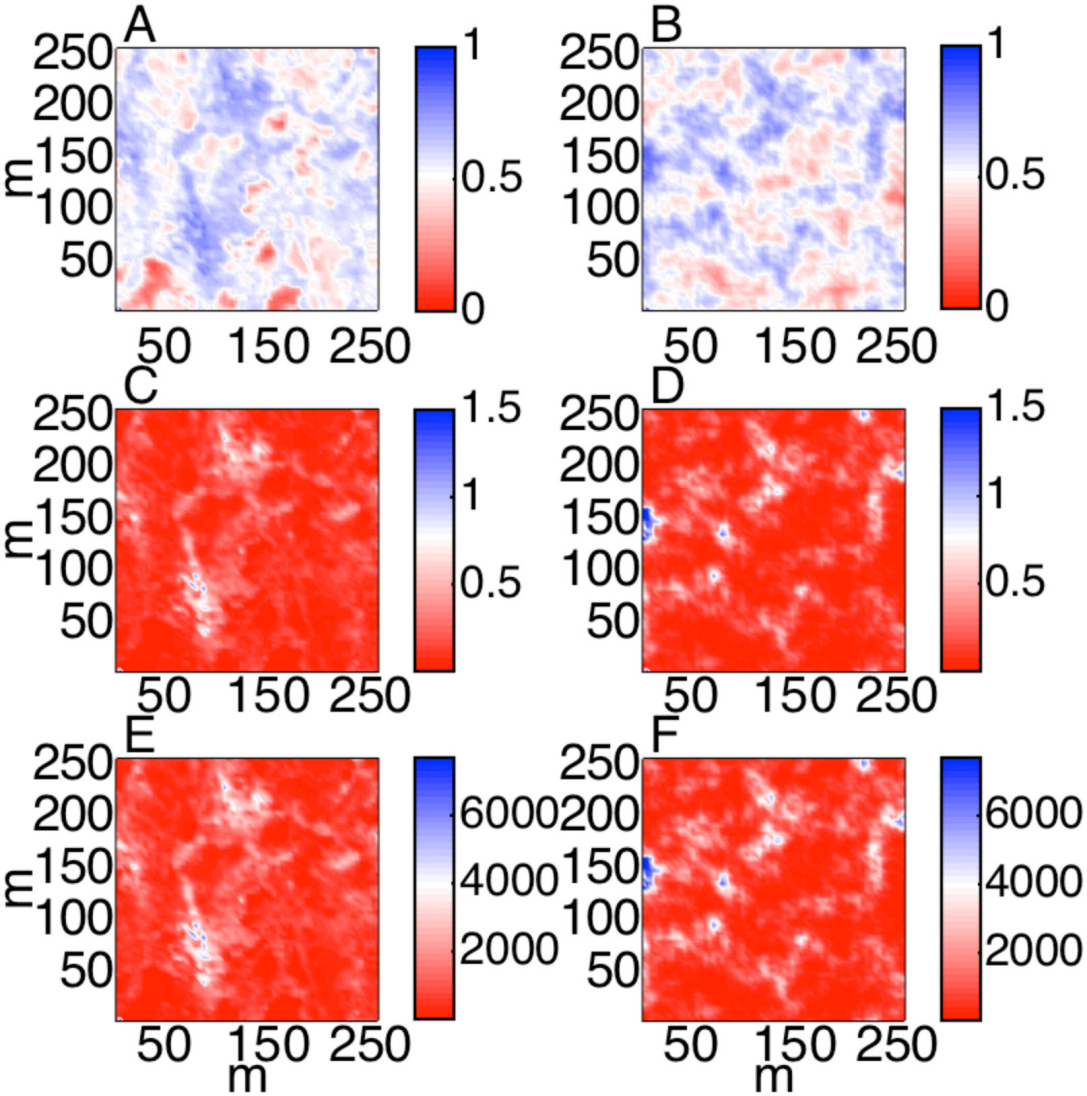
NDVI maps from observations (A) and simulations using two-dimensional Tikhonov Regularization with a Lagrange multiplier γ = 100.85 (B). LAI maps that result from the observed (C) and simulated (D) NDVI values. Corresponding maps of the June-July GPP simulated by PLIRTLE for observations (E) and simulations (F) in g C per growing season per 16 m^2^ pixel.

The observed NDVI map (Figs 1A and 5A) and the single realization of a downscaled NDVI map with representative *γ*, here *γ* = 10^0.85^ (Fig 5B), can be converted to LAI using the equations in [27] (Fig 5C and 5D), and used to drive the PLIRTLE model with measured meteorological input [21,30] to estimate GPP (Fig 5E and 5F). Using this approach we arrive at a GPP estimate of 3488 kg C during the June-July growing season for the study area from the observed NDVI map and 3427 kg C during the growing season for the study area from the simulated NDVI map, a difference of about 2%. This landscape-level GPP estimate from observations is equivalent to an average of 0.92 g C m^-2^ day^-1^ during the growing season (0.90 g C m^-2^ day^-1^ from simulated NDVI), not dissimilar to the average of 0.95 g C m^-2^ day^-1^ in a nearby tundra ecosystem calculated from eddy covariance observations [35].

**Fig 6:**
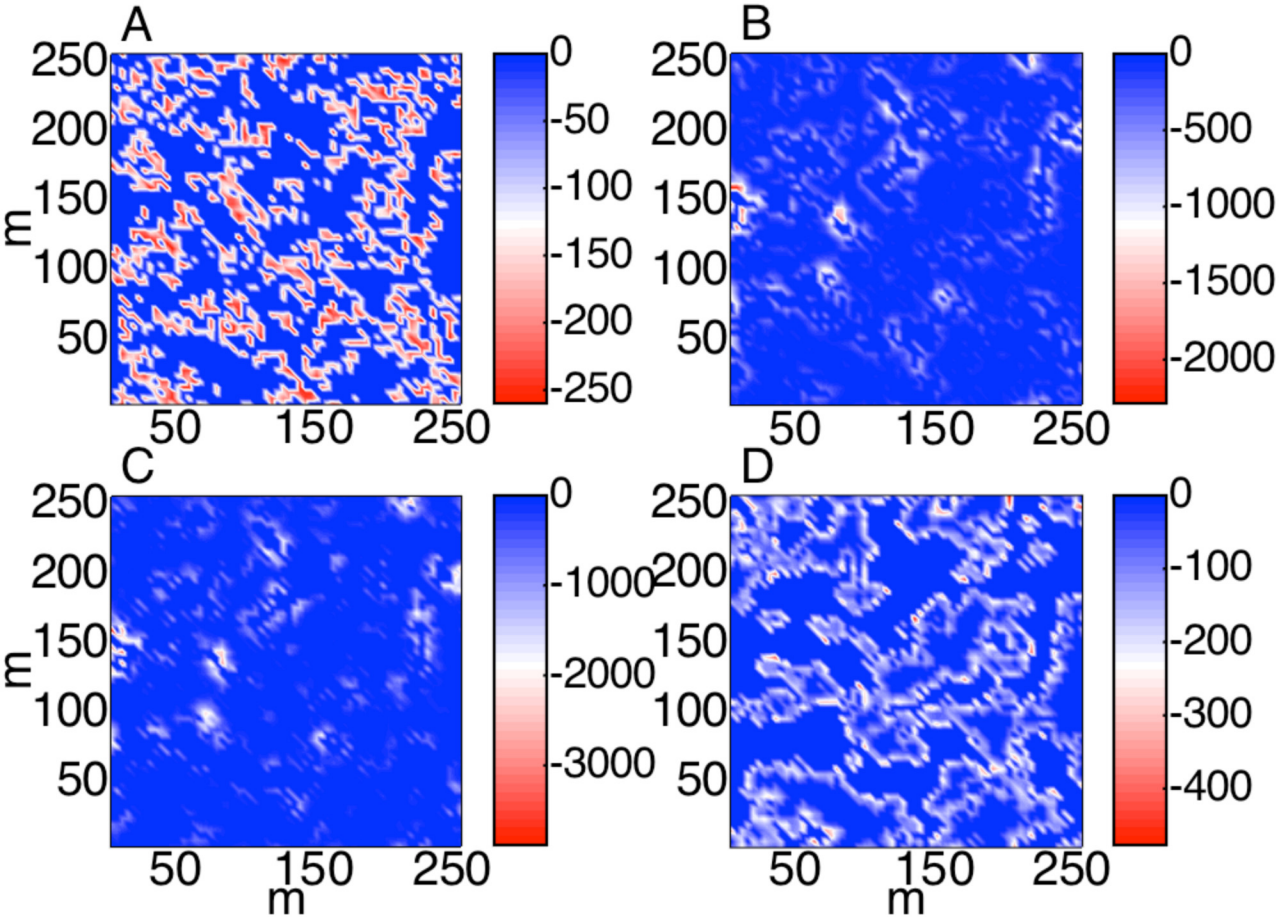
The difference between June-July growing season gross primary productivity (GPP) in g C per growing season per 16 m^2^ pixel that result from NDVI maps simulated using two-dimensional Tikhonov Regularization with Lagrange multiplier *γ* = 10^0.85^ (Fig 5F) and GPP maps that result from estimating patch edge using Method 1 (A), 2 (B), 3 (C), and 4 (D) as described in the text. Please note that the scale of each subplot is unique.

**Fig 7:**
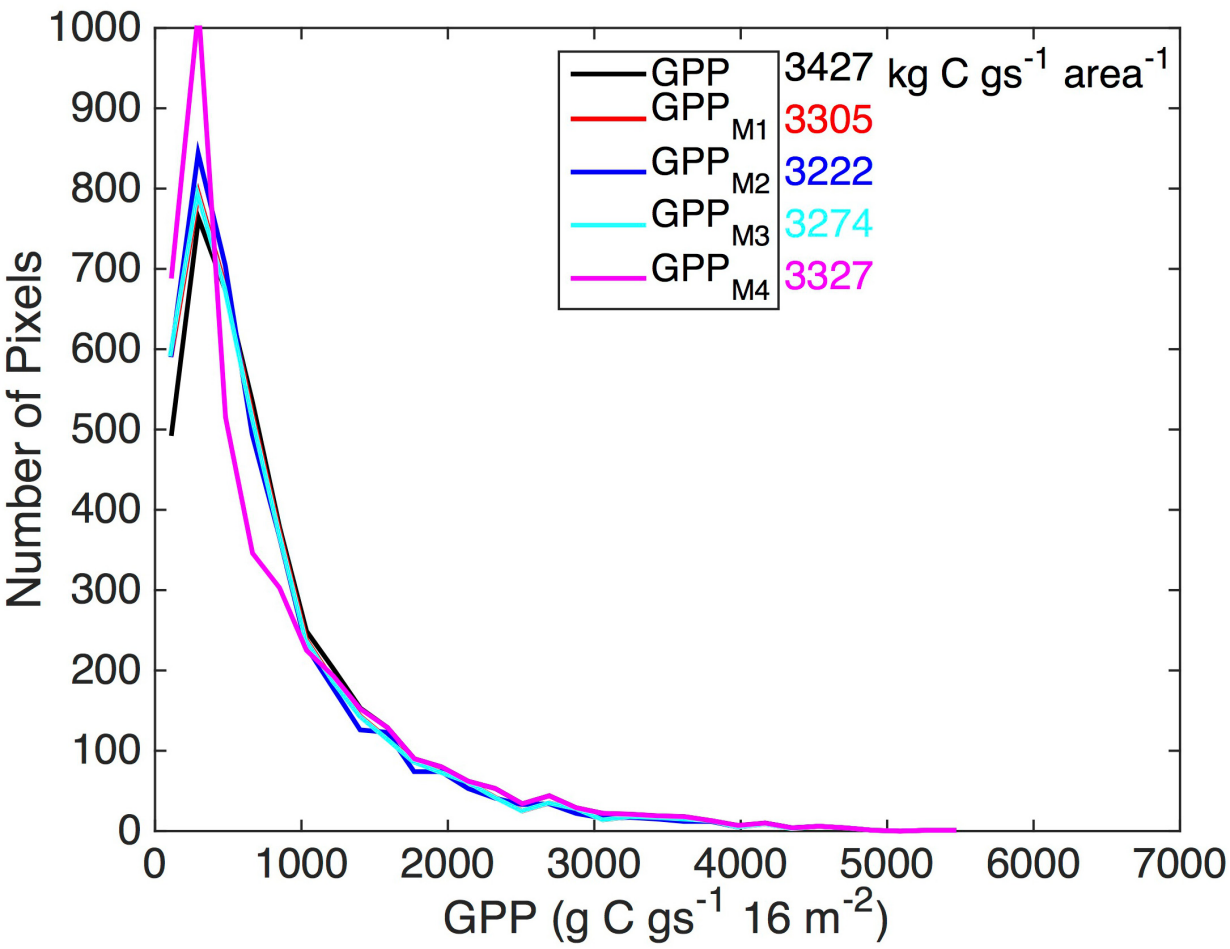
Histograms of gross primary productivity that result from an NDVI maps simulated using two-dimensional Tikhonov Regularization with Lagrange multiplier *γ* = 10^0.85^ (Fig 5F) and different methods for estimating patch edge as described in the text. The landscape-level GPP that results from the different approaches is presented using the same color scheme as the legend.

The different methods for simulating edge resulted in different patters of GPP reduction (Fig 6). Histograms of per-pixel GPP and the growing season sums of landscape-level GPP for this single iteration of 2DTR are shown in Fig 7. Method 1, which essentially reduced GPP in pixels that took values near the mean NDVI value of 0.54, resulted in frequent minor reductions in GPP. This is in contrast to the methods that use gradient approaches (Methods 2 and 3), which demonstrate frequent sharp reductions in GPP in areas where GPP is large, i.e. toward patch centers. The spatial patterns of GPP reduction for Method 4, where edge was ascertained by rounding NDVI to the nearest integer, are similar to Method 1. Upon one thousand iterations of 2DTR, growing season GPP estimated using Methods 1 and 4 was 3380 ± 41 kg C and 3387± 41 kg C, respectively, or 3% lower than GPP derived using NDVI observations. GPP estimated using Method 2 was 8% lower than GPP derived using NDVI observations (3197 ± 50 kg C during the growing season), and Method 3 averaged 11% lower than GPP derived using NDVI observations (3106 ± 96 kg C during the growing season). A 9% GPP reduction (i.e. a 30% reduction for 30% of pixels) would be expected if edge were to be assigned randomly.

## Discussion

### Simulating subgrid statistics

The 2DTR approach explored here is able to capture the dominant modes of spatial variability of complex subgrid information (Figs 2 and 4) if the range of the semivariogram of subgrid elements [29], related to *γ* (Fig 3), is known. Further, 2DTR as applied here is challenged by landscapes with multiple modes of variability in space; for example the landscapes simulated in this example cannot capture the minor peak in the radially-averaged power spectrum of observed NDVI at *ca*. 15–20 m (Fig 4), but landscapes generated with multiple values of *γ* could be combined if simulating multiscale variability is of importance.

In many remote sensing applications, only the mean pixel value (assuming minimal bias) of an observable like the NDVI is known, and the total subpixel variance and subgrid statistics are unknown. At a minimum, the variance of a remotely-sensed pixel, and preferably also spatial statistics and higher order statistics like skewness [21] or the statistics of non-Gaussian distributions, should be reported if possible. Whereas our study only considered a spatial grain of 4 m, simulating the statistics of smaller-scale features is possible if their characteristic dimensions are known; such subgrid information may be critical for example for modeling methane flux [37,38]. Many tundra ecosystems exhibit patterned ground features due that are created by freeze/thaw dynamics and vary on characteristic spatial scales on the order of 0.5 to 3 m in the case of frost boils [39–42] or a few meters to hundreds of meters in the case of ice-wedge polygons [41,42], and these attributes can be simulated using 2DTR. Other recommended characteristic pixel sizes depend on ecosystem type and application; for example, Rahman et al. [29] found that a pixel size of 6 m or less was optimal for characterizing the variability of grassland and chapparal in the Mediterranean climate zone using hyperspectral remote sensing.

Another emerging question for subgrid scaling, although long an active area of ecological research, is the extent and characteristics of vegetation patch edges [43–45]. Edges between patches in the study area have lower GPP per unit LAI [24] and comprise a nontrivial proportion of the study landscape. The most effective method to simulate where edge occurs is unclear without a detailed map of the locations that have the characteristics of edge. As a consequence, we simply tested the implications of four different assumptions regarding the location of edge in relation to vegetation patches as approximated by the spatial distribution of NDVI. Some methods for simulating vegetation patch edge reduced landscape-level GPP more than others, although the difference among approaches was on the order of 10% or less (Figs 6 and 7). For the case of simulating the functioning of patterned ground in tundra, Cresto Aleina et al. [37] introduced a tunable parameter that adjusted the ratio of polygon rims and centers based on ground and aircraft observations [46]. Ice-wedge features have been successfully characterized using 0.46 m remote sensing observations from WorldView-2 [47], but it remains less clear how to characterize the more subtle transition zones between vegetation patches in tundra ecosystems that are not dominated by such striking features using remotely-sensed data.

### Relationship to the Maximum Entropy Principle

The Tikhonov Regularization approach explored here is a two-dimensional representation of a classic mathematical procedure for dealing with ill-posed problems. A general solution for obtaining the least biased estimate of unknown information was tackled by Jaynes (1957) in his classic Maximum Entropy Principle in which he states, “…in making inferences on the basis of partial information, we must use that probability distribution which has the maximum entropy subject to whatever is known”. The most likely pdf, given available information, is that which maximizes information entropy subject to known constraints. These constraints represent logical, physical and biological bounds on the underlying pdf, similar to those used here. Maximum Entropy applications in ecology and geoscience to date include scaling biological diversity and species abundance [48–50], species range modeling [51–54], remote sensing image analysis [55], and ascertaining the most likely state of the climate system [56] as an extension of the Maximum Entropy Production hypothesis [57]. Our 2DTR approach does not explicitly maximize entropy, but 2DTR is typically found to give comparable results to more computationally expensive maximum entropy regularisation procedures [58,59]. We are motivated by the need to produce a practical tool for subgrid scaling in ecology and geoscience, and 2DTR represents a fast analytical alternative to numerical maximum entropy regularization solvers. In particular, once the inverse term in (1) has been computed and stored, a large number of scenes can be generated quickly.

### Additional applications of 2DTR in functional ecology and geoscience

Inferring surface pattern using 2DTR may also be used for tackling other aspects of the subgrid scaling problem [60] including studies on large eddy simulation and multiscale biosphere-atmosphere coupling [61]. Inferring pattern is also important for simulating highly nonlinear biogeochemical fluxes like that of methane which often requires a detailed knowledge of the microtopography and species composition of the land surface with respect to water table height [62,63]. Methods to infer pattern make up one part of the grand scaling challenge in remote sensing, ecology and biogeochemistry, and 2DTR is but one of many approaches that have been developed to address issues of scale in Ecology and Earth Science [9,10,13,14], albeit one that assumes minimal prior information. We recommend future comparisons amongst multiple landscape generation methods and envision that 2DTR will be a useful contribution in the Earth scientists’ toolkit for addressing scaling challenges.

## Conclusions

We simulated the fine scale (4 m) spatial statistics of the NDVI of a tundra landscape using 2DTR from coarse-scale (250 m) mean NDVI. Results demonstrate that there is a functional relationship between the range of the experimental variogram and the value of *γ* that describes the strength of the assumption that adjacent fine-scale pixels are similar. Landscapes simulated using 2DTR with the value of *γ* that corresponds to the range of the experimental semivariogram derived from observations captured the dominant mode of spatial variability of observations as revealed by radially-averaged power spectra. We demonstrate that 2DTR is applicable for simulating subgrid statistics in a tundra landscape and suggest that this approach may find application in creating synthetic landscapes for ecological and Earth systems applications.
